# Expanding the swimmer’s itch pool of the Benelux: a first record of the neurotropic *Trichobilharzia regenti* and potential link to human infection

**DOI:** 10.1186/s13071-024-06218-4

**Published:** 2024-03-13

**Authors:** Ruben Schols, Nathalie Smitz, Ann Vanderheyden, Tine Huyse

**Affiliations:** 1 Department of Biology & BopCo, Royal Museum for Central Africa, Tervuren, Belgium; 2 Laboratory of Aquatic Biology, KU Leuven, Campus Kortrijk, Kortrijk, Belgium; 3 BopCo, Royal Belgian Institute of Natural Sciences, Brussels, Belgium

**Keywords:** Trematode infections, Schistosomatidae, Zoonoses, Trichobilharzia, Dermatitis, Snail-borne diseases, Vector-borne diseases, Climate change, DNA barcoding

## Abstract

**Background:**

Swimmer's itch, an allergic contact dermatitis caused by avian and mammalian blood flukes, is a parasitic infection affecting people worldwide. In particular, avian blood flukes of the genus *Trichobilharzia* are infamous for their role in swimmer’s itch cases. These parasites infect waterfowl as a final host, but incidental infections by cercariae in humans are frequently reported. Upon accidental infections of humans, parasite larvae will be recognized by the immune system and destroyed, leading to painful itchy skin lesions. However, one species, *Trichobilharzia regenti,* can escape this response in experimental animals and reach the spinal cord, causing neuroinflammation. In the last few decades, there has been an increase in case reports across Europe, making it an emerging zoonosis.

**Methods:**

Following a reported case of swimmer’s itch in Kampenhout in 2022 (Belgium), the transmission site consisting of a private pond and an adjacent creek was investigated through a malacological and parasitological survey.

**Results:**

Six snail species were collected, including the widespread *Ampullaceana balthica*, a well-known intermediate host for *Trichobilharzia* parasites. Shedding experiments followed by DNA barcoding revealed a single snail specimen to be infected with *T. regenti,* a new species record for Belgium and by extension the Benelux. Moreover, it is the most compelling case to date of the link between this neurotropic parasite and cercarial dermatitis. Additionally, an Echinostomatidae sp. and *Notocotylus* sp. were isolated from two other specimens of *A. balthica*. However, the lack of reference DNA sequences for these groups in the online repositories prevented genus- and species-level identification, respectively.

**Conclusions:**

The presence of *T. regenti* in Belgium might have severe clinical implications and its finding highlights the need for increased vigilance and diagnostic awareness among medical professionals. The lack of species-level identification of the other two parasite species showcases the barcoding void for trematodes. Overall, these findings demonstrate the need for a Belgian framework to rapidly detect and monitor zoonotic outbreaks of trematode parasites within the One Health context.

**Graphical Abstract:**

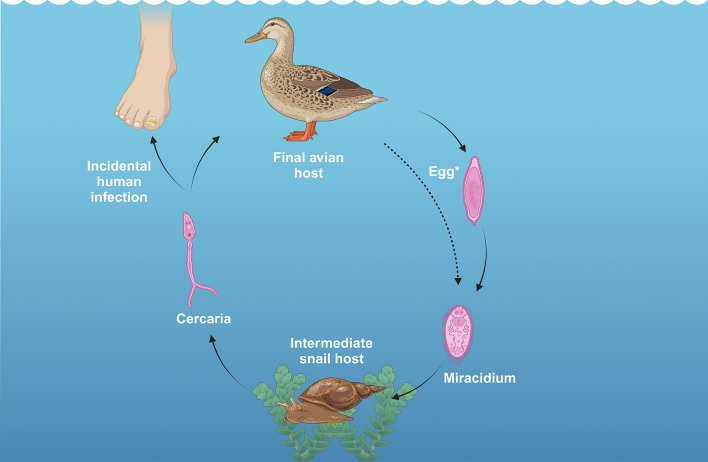

**Supplementary Information:**

The online version contains supplementary material available at 10.1186/s13071-024-06218-4.

## Background

Cercarial dermatitis, or swimmer’s itch, is caused by mammalian and avian schistosomes (Trematoda: Schistosomatidae) [1]. The most important schistosome species belong to the genus *Schistosoma,* affecting more than 200 million people worldwide, with the largest burden in Sub-Saharan Africa [2]. Nevertheless, cercarial dermatitis outbreaks across the world are rarely attributed to *Schistosoma* spp. [3]. Instead, outbreaks in temperate climate regions, such as the USA or Europe, are usually attributed to species belonging to the closely related *Trichobilharzia* genus [3]. This genus is one of seven genera of bird schistosomes described in Europe, which mostly parasitize ducks, geese, or swans [4, 5].

**Fig. 1:**
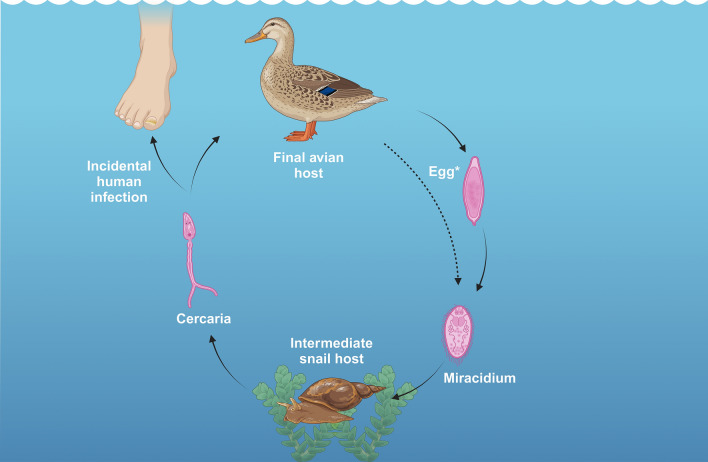
The *Trichobilharzia* spp. lifecycle. The lifecycle usually involves pulmonate freshwater snails as intermediate hosts and waterfowl as final hosts. Human infections occur when cercariae incidentally penetrate human skin during water-bound activities, potentially causing swimmer’s itch. *The eggs of nasal *Trichobilharzia,* such as *Trichobilharzia regenti,* already hatch into miracidia in the nasal tissue of the final host, leaving the nasal cavity while the host drinks or feeds (dotted line). This is in contrast to the lifecycle of visceral *Trichobilharzia*, whereby miracidia emerge from eggs following contact with water. Created with BioRender.com

Bird schistosomes of the genus *Trichobilharzia* are ubiquitous trematode flatworms that infect pulmonate freshwater snails as intermediate host and waterfowl as final hosts (Fig. 1). *Radix* spp. and *Lymnaea stagnalis* Linnaeus, 1758 are most frequently reported as suitable intermediate snail hosts for *Trichobilharzia* species, yet a plethora of other snails have been described to transmit avian schistosomes [3, 4]. The many snail hosts, some with a high abundance and wide distribution, contribute to the near-global geographic range of bird schistosomes [1]. Adult *Trichobilharzia* parasites inhabit either the visceral venous system or nasal tissue of waterfowl, depending on the species. Firstly, visceral species such as *Trichobilharzia szidati* Neuhaus, 1952 and *T. franki* Müller & Kimmig, 1994 are located in the intestinal and other veins and produce eggs that enter the freshwater environment through feces. Upon contact with fresh water, these eggs hatch into miracidia and start swimming to localize a suitable snail host. Secondly, adults of the nasal species, such as *Trichobilharzia regenti* Horák, Kolářová & Dvořák, 1998 are located in the nasal veins and lay eggs that hatch into miracidia in the nasal tissue, which can leave the final host in search of an intermediate snail host when it feeds or drinks. Upon snail infection, the miracidia will further develop and reproduce asexually, after which the cercarial stage will leave the snail. Hundreds to thousands of cercariae are released on a daily basis [6, 7], which in turn infect the final bird host, where they can develop into adults and start egg production. However, when encountering a human, the cercariae attach to the skin and preferentially attempt to penetrate through wrinkles or hair follicles [1]. Cercarial dermatitis arises due to an immune response that worsens with repeated exposure, effectively blocking further migration into the human body [1, 8]. However, upon first exposure of immunocompetent or in primary and challenge infections in immunodeficient murine model systems, some schistosomula survive and manage to migrate further in the body [9, 10]. The inability to stop these parasites from migrating throughout the mouse’s body is especially impactful for the neurotropic *T. regenti*, whereby 10% of the infecting larvae may reach the peripheral nervous system [9, 10].

Swimmer’s itch is considered an emerging cosmopolitan condition affecting human health and leading to economic losses due to the closing of recreational areas [4, 11, 12]. Soldánová et al. [4] review a broad range of ecological factors that can drastically affect swimmer’s itch incidence. Increased nutrient concentrations boost snail abundance, snail growth, and bird visits, and consequently increase the trematode parasite biomass. In addition, higher temperatures, such as summer heatwaves, are often linked to cercarial dermatitis outbreaks, as swimming time, parasite development, and cercarial emergence all increase. Coinfections may also affect swimmer’s itch incidence as intramolluscan competition can drive trematode community composition [4, 13, 14].

The genus *Trichobilharzia* consists of more than 35 species [15], and two have been linked to outbreaks of cercarial dermatitis across Europe: the visceral *T. szidati* and *T. franki* [8, 16–18]. Five additional *Trichobilharzia* species have been identified as potential agents of cercarial dermatitis in Europe: *T. physellae* Talbot, 1936 [19], *T. mergi* Kolářová, Skírnisson, Ferté, and Jouet, 2013 [20], *T. anseri* Jouet, Kolářová, Patrelle, Ferté, and Skírnisson, 2015 [21], *T. salmanticensis* Simon-Vicente and Simon-Martin, 1999 [22], and *T. regenti* [23]. The first scientifically recorded outbreak of swimmer’s itch in Belgium occurred in 2012 and was attributed to *T. franki* infections [16]. The present study focuses on a private pond located in central Belgium following complaints of extremely painful and itchy skin lesions after swimming on the weekend of 17 June 2022. We set out to (1) map snail and trematode diversity within the respective water systems and (2) ascertain the possible cause of the reported swimmer’s itch.

## Methods

### Snail collection

**Fig. 2:**
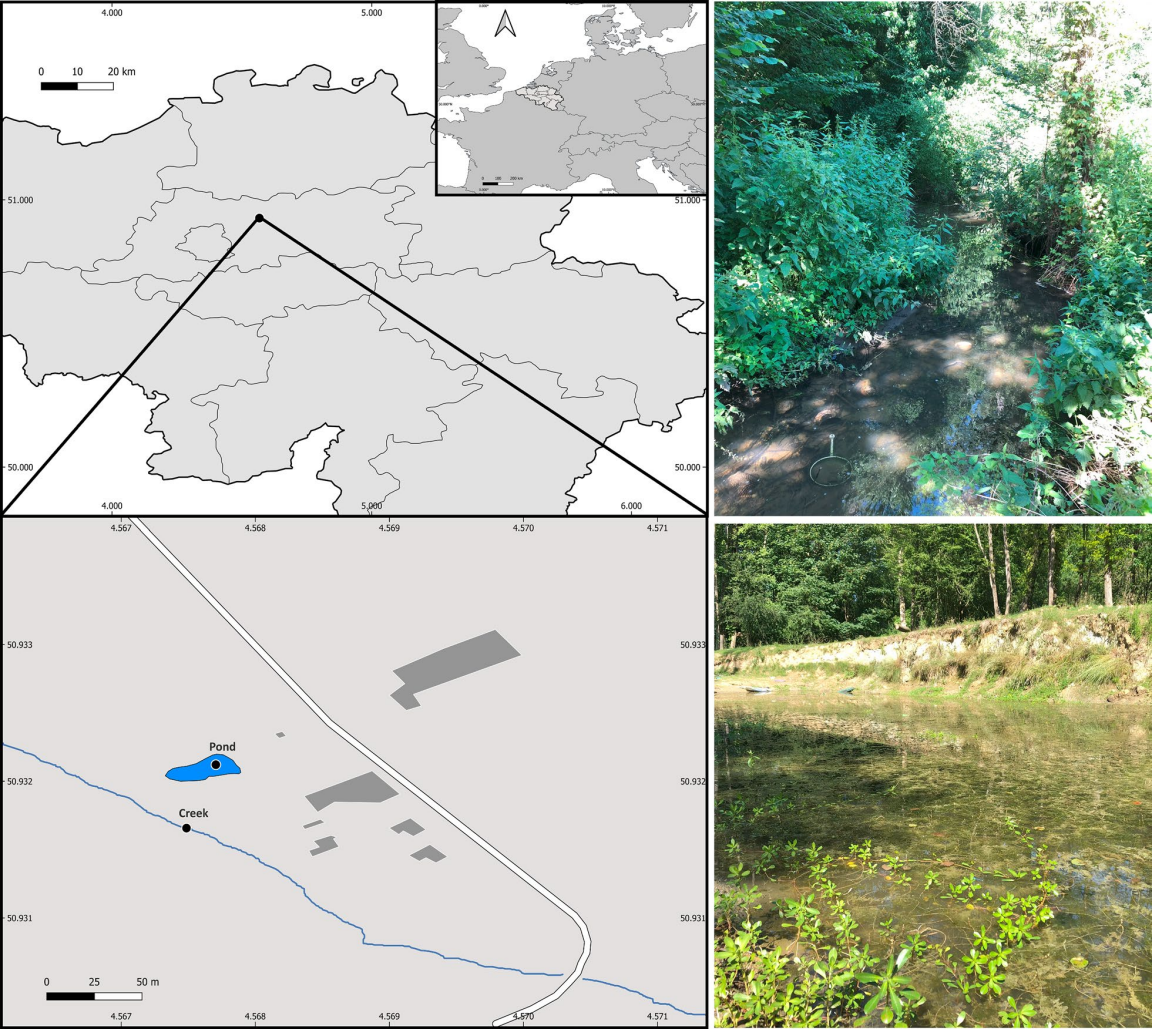
Sampling area in Kampenhout (Flemish Brabant province; Belgium). The dots indicate the location of Kampenhout within Belgium, as well as the creek and pond sites on the second inset. The top right and bottom right pictures illustrate the creek and pond site, respectively

Snails were collected on 29 June 2022, 11 days after the onset of swimmer’s itch symptoms, from two sites in the Flemish part of Belgium (pond coordinates: Lat.: 50.932121, Long.: 4.567706; creek coordinates: Lat.: 50.931657, Long.: 4.567487; Fig. 2) using a scooping net and protective gloves. The sampling was restricted to the margins of the pond due to the steep bottom inclination: up to two meters from the water’s edge. The content of the scooping net, aquatic vegetation, and any substrate were inspected for the presence of freshwater snails. Captured snails were transported alive in containers with water from the sites of origin to the Royal Museum for Central Africa (RMCA) in Tervuren (Belgium) for further analyses.

### Shedding experiment

Upon arrival at RMCA, all snails were sorted per morphotype and per site and placed individually in multiwell cell culture plates with water from the site of origin. A subset of the *Lymnaea stagnalis* specimens had to be pooled in a larger container due to their large size and high abundance. Each well was inspected 1-h post-isolation at 4 pm to ascertain afternoon cercarial shedding. If only one cercaria was found, we attributed it to contamination of the source water, but none were found. The snails were then kept in the dark at room temperature until the next day. In the early morning, all plates were visually inspected with a stereo microscope to verify whether nocturnal shedding had taken place. Subsequently, bright artificial light exposure started at 7 am and blinds were opened to allow natural light to illuminate the room further. All wells were inspected at 8 am, 10 am, and 12 pm for cercariae. All snails were sacrificed at 12 pm. Released cercariae were stored in a separate tube with 70% ethanol for optimal preservation. All shedding snails were further stored individually, while uninfected snails were pooled per morphotype per site. All samples were stored in 70% ethanol at + 4 °C until further processing.

### Morphological identification of snails and larval trematodes (cercariae)

Snails were morphologically identified on the basis of external shell characteristics using the Field Guide to Slugs and Mussels [24]. Isolated cercariae were morphologically classified to cercarial type level on the basis of the identification guide of Frandsen and Christensen [25].

### Digitalization

Following the methodology described in Brecko et al. [26], high-resolution photographs were captured for all snail morphotypes using the ZereneR stacker software (T2019-10-07-1410) in a focus stacking system. Subsequently, the resulting images underwent post-processing in Adobe Acrobat Photoshop (version 23.1.1). This involved several modifications, such as eliminating the support structure on which the snail was mounted, merging the forward and reverse orientations into a single image, and including an appropriate scale bar.

Cercariae isolated from each snail were poured into a watch glass with 70% ethanol and visually inspected with a stereomicroscope for different morphotypes. Four specimens per morphotype per snail were isolated for DNA extraction, one of which was first placed on a microscope glass in sterile milli-Q water with a cover glass. The BMS camera (model no.: XFCAM1080PHD) and associated BMS_pix3 software (version 3.7.8942.20170412) were then used to photograph each specimen at all focal depths while manually adjusting the specimen’s position. The resulting images were stacked with the ZereneR stacker software and post-edited with Adobe Acrobat Photoshop (version 23.1.1) for the removal of background noise and the addition of an appropriate scale bar.

### DNA extraction and Sanger sequencing

DNA was extracted from the internal organs of the snails (excluding the head and foot) with the E.Z.N.A. Mollusc DNA Kit (OMEGA bio-tek, Inc.), according to the manufacturer’s guidelines and following Schols et al. [27], with an elution volume of two times 75 µl. DNA was extracted from the entire individual cercaria using a lysis buffer containing proteinase *K* following the methodology outlined in Zietara et al. [28], resulting in a final volume of 20 µl. The remaining soft tissue parts (head and foot) of the snail specimens together with their respective shells and dried DNA extracts were stored at the Royal Belgian Institute of Natural Sciences (collection codes: RBINS:IG34767:INV311000).

Two snail and three trematode genetic markers were amplified using specific primer combinations. Part of the mitochondrial cytochrome *c* oxidase subunit I (COI) [29] for all snails, and of the rDNA region spanning the Internal Transcribed Spacer 2 (ITS2) marker for the *Stagnicola* snails [30, 31], were targeted for species identification using the primer combinations as displayed in Additional file 1: Tables S1, S2. Trematode-specific primers were used for the amplification of partial regions of the COI [32, 33], the 18S ribosomal RNA (18S) [32, 34], and the Internal Transcribed Spacer 2 (ITS2) [33] (Additional file 1: Tables S1, S2).

All amplifications, except for the ITS2 in *Stagnicola* snails, were performed in 20 µL reaction mixtures, including 2 µL of DNA template, 2 µL of 10X buffer, 1.5 mM MgCl_2_, 0.2 mM dNTP, 0.4 µM of each primer, and 0.03 units/µL of Platinum^™^ Taq DNA Polymerase (InvitrogenTM, Waltham, MA, USA). The cycling conditions were as displayed in Additional file 1: Tables S1, S2. Furthermore, the ITS2 region in *Stagnicola* sp. specimens was amplified following Schols et al. [35], using the Qiagen^™^ Taq DNA polymerase kit and the cycling conditions as displayed in Additional file 1: Tables S1, S2. Polymerase chain reaction (PCR) products and negative controls were checked on a 2% agarose gel using a UV transilluminator and the MidoriGreen^™^ Direct (NIPPON Genetics Europe, Dueren, Germany) method. Positive amplifications were subsequently purified using the ExoSAP-IT^™^ protocol (following manufacturer’s instructions) and sequenced in both directions using the BigDye^®^ chemistry (Macrogen™).

### Analyses

Assembled bidirectional nucleotide strands were trimmed, corrected, and translated into amino acids to check for stop codons (if coding region) using Geneious Prime^®^ 2019.2.3 (Biomatters Ltd., Auckland, New Zealand). A consensus sequence was generated for each DNA sequence and each specimen. Subsequently, consensus sequences were compared using the BOLD Identification System, with the Species Level Barcode Records option (www.boldsystems.org), and the Basic Local Alignment Search Tool (BLAST) of GenBank (https://blast.ncbi.nlm.nih.gov/Blast.cgi).

## Results

**Fig. 3:**
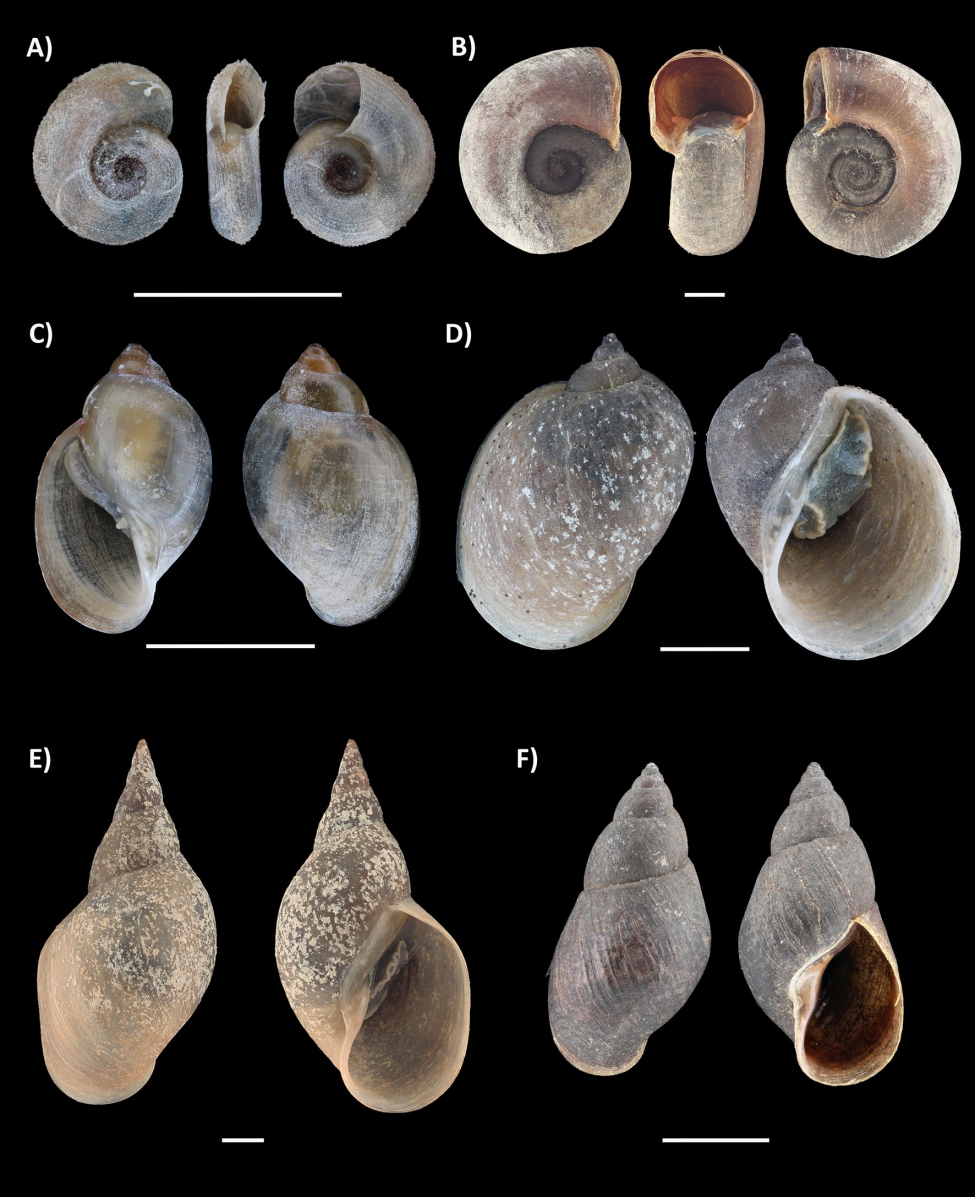
Different snail species collected at the pond and creek sites in Kampenhout (Flemish Brabant province; Belgium). **A** *Gyraulus albus*, **B** *Planorbarius corneus*, **C** *Physella acuta*, **D** *Ampullaceana balthica*, **E** *Lymnaea stagnalis*, and **F** *Stagnicola fuscus*. Scale bar represents 5 mm in each picture

From the 159 snail specimens collected across the sampling area, six different snail species were identified: *Gyraulus albus* O. F. Müller, 1774; *Planorbarius corneus* Linnaeus, 1758; *Physella acuta* (Draparnaud, 1805); *Lymnaea stagnalis, Ampullaceana balthica* (Linnaeus, 1758); and *Stagnicola fuscus* Pfeiffer, 1821 (Fig. 3). The latter species was only retrieved from the creek site, together with *P. corneus*. The other four species occurred in the main pond (Table 1).

**Table 1:** Snail species and counts for both sampling sites, as well as the GenBank pairwise identity ranges expressed in percentages based on the COI sequences, with an exception for *Stagnicola fuscus*, for which ITS2 was also used

Snail species	Pond site (*N*)	Creek site (*N*)	Sequenced specimens (*N*)	GenBank pairwise identity (accession numbers)
*Ampullaceana balthica*	11 (3)	0	11	COI: 100–99.8% (MW801451, KP242705)
*Lymnaea stagnalis*	108	0	4	COI: 100% (ON653340)
*Stagnicola fuscus*	0	3	2 (COI); 1 (ITS2)	ITS2: 100% (HG931948)
*Physella acuta*	30	0	2	COI: 100% (OW485645, LC582932)
*Planorbarius corneus*	2	4	4	COI: 100–99.4% (MT862415, AY227370)
*Gyraulus albus*	1	0	1	COI: 99.4% (KC495835)

The number of snails releasing cercariae is listed in between parentheses

All morphological snail species identifications were validated using COI barcodes, except for *S. fuscus*, for which the investigation of the ITS2 marker was necessary to discriminate pure *S. fuscus* from *S. fuscus* × *S. palustris* hybrids [31] (GenBank pairwise identity ranges displayed in Table 1). ITS2 sequencing was attempted on both COI-barcoded *Stagnicola* specimens, one of which was eventually of sufficient quality for identification. This *Stagnicola* specimen was identified as pure *S. fuscus* on the basis of the ITS2 marker. All generated snail-specific sequences were deposited on GenBank, with accession numbers: COI: PP203049- PP203072; ITS2: PP203073.

**Fig. 4:**
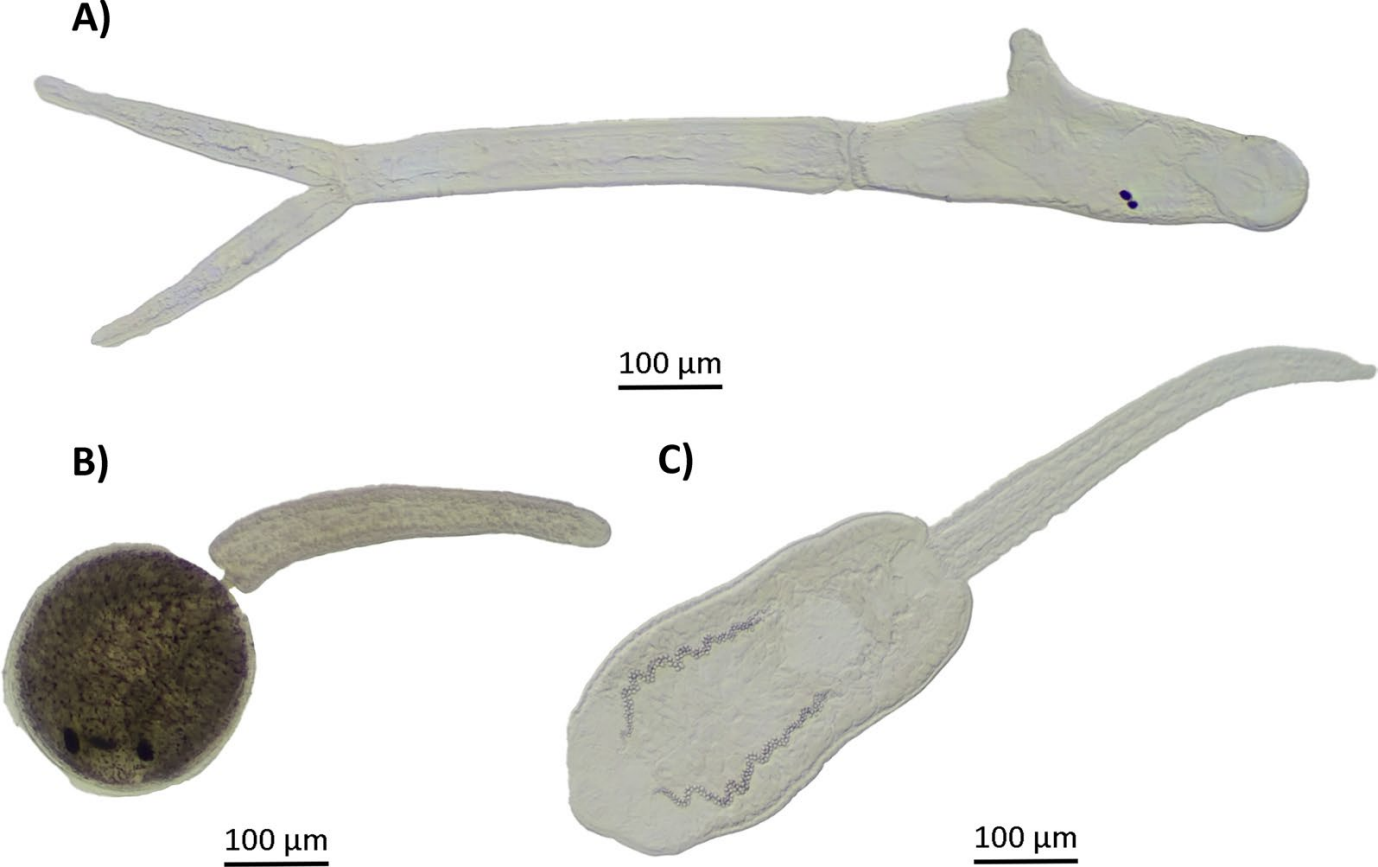
High-resolution pictures of cercariae released by three *Ampullaceana balthica* specimens during the shedding experiment. **A** *Trichobilharzia regenti*, **B** *Notocotylus* sp., and **C** Echinostomatidae sp.

Three specimens of *A. balthica* released cercariae during the shedding experiment, each one of them releasing one unique cercarial morphotype (Fig. 4). Details on the BLAST results are listed for all three markers in Table 2. Cercariae of the specimen V_RAD_4 were of the brevifurcate-apharyngeate distome type; the DNA sequences (18S, ITS, and COI) were 99.9–100% identical to GenBank reference sequences of *Trichobilharzia regenti* from the Czech Republic [ITS: 99.9% (OX235267); 18S: 100% (AY157218)] and France [COI: 100% (HM439500)] (Fig. 4A, Table 2). Snail specimen V_RAD_1 showed an infection with monostome type cercariae identified as *Notocotylus* sp. Diesing, 1839 on the basis of all three DNA markers (Fig. 4B, Table 2). For V_RAD_3, echinostome type cercariae were collected. The genetic identification results were ambiguous as the nuclear markers 18S and the ITS region seem to indicate either an *Echinoparyphium recurvatum* (von Linstow, 1873) Lühe, 1909 or an *Echinostoma revolutum* (Fröhlich, 1802) Looss, 1899 infection, while the COI sequence had the highest pairwise identity of 87.8% with *Echinoparyphium* sp., which dropped to 82% for *Echinostoma revolutum* (Table 2). Consequentially, the identification of this species can only be ascertained to the family level, being the Echinostomatidae (Looss, 1899) (Fig. 4C, Table 2). All generated trematode sequences were deposited in GenBank, with accession numbers: COI: PP232100- PP232102; ITS: PP274989- PP274991; 18S: PP232103- PP232105.

**Table 2:** Cercariae trematode identification results based on the investigation of the nuclear (18S rDNA and ITS region) and mitochondrial (COI) DNA sequences

Trematode sample	18S rDNA	ITS region	COI mtDNA	Species identification conclusion
V_RAD_1	1043 bp—100% (AJ287547)—*Notocotylus* sp.	1240 bp—97.7% (JQ766940)—*Notocotylus malhamensis*	419 bp—99.5% (MH369327)—*Notocotylus* sp.	*Notocotylus* sp. (Fig. 4B)
V_RAD_3	1050 bp—100% (OP627676)—*Echinostoma revolutum*	950 bp—100% (OP627676)—*Echinostoma revolutum* / 100% (AY168931)—*Echinoparyphium recurvatum*	890 bp—87.8% (MH369225)—*Echinoparyphium* sp. (82% (OR030109)—*Echinostoma revolutum*)	Echinostomatidae sp. (Fig. 4C)
V_RAD_4	1040 bp—100% (AY157218)—*Trichobilharzia regenti*	1070 bp—99.9% (OX235267)—*Trichobilharzia regenti*	800 bp—100% (HM439500)—*Trichobilharzia regenti*	*Trichobilharzia regenti* (Fig. 4A)

The table presents the amplicon length (bp), BLAST pairwise identity (%), and best match together with the GenBank accession number per sample and per sequence. The last column reports the resulting identification on the basis of the compilation of all BLAST outputs

## Discussion

This study reports on the first record of *Trichobilharzia regenti* in Belgium, while also investigating the local pulmonate freshwater snail and trematode biodiversity in two bodies of water located in Kampenhout (Belgium). All but one snail species collected in this study are native to Belgium. *Physella acuta* is an invasive snail originating from North America that has been reported in Belgium since 1869 [36]. It is a globally invasive species with records ranging from Argentina and South Africa to Australia and the Netherlands [37]. No larval trematodes were released from this snail species, which is consistent with the enemy-release hypothesis that describes the observation that many exotic species have a lower parasite load outside their native range [37]. In contrast, cercariae of three trematode species were released from the native *A. balthica*. The trematode species of the family Echinostomatidae infect its final vertebrate host through trophic interactions, i.e., when infected secondary intermediate hosts such as snails, fish, or amphibians are consumed by the final host. It has been linked to human infections in Africa, Asia, and Europe, although most case reports stem from Asia due to the routine consumption of raw fish [38]. The second species we encountered, *Notocotylus* sp., belongs to the monostome type cercariae, which rapidly encyst to form metacercariae on nearby substrates [39]. The exact lifecycle of most *Notocotylus* sp. is not fully known but final avian or rodent hosts become infected upon ingestion of these metacercariae during foraging [39]. Finally, the third trematode species was identified as *T. regenti*. At present, *T. regenti* is the second *Trichobilharzia* species reported in Belgium, after the swimmer’s itch outbreak caused by *T. franki* in 2012 in the Plate Taille Lake (approximately 90 km south of our sampling location) [16].

*Trichobilharzia regenti* has already been described from snails and birds in several European countries (See [11, 23, 40–46]), and has been suggested as a potential cause of swimmer’s itch. However, until now, *T. regenti* always co-occurred with other agents of swimmer’s itch in the studied transmission sites. As a result, it was difficult to determine which of the *Trichobilharzia* species was responsible for the reported dermatitis. Since we only found *T. regenti* in our study, it more strongly supports *T. regenti* as a causative agent of swimmer’s itch. *Trichobilharzia regenti* may potentially be the most dangerous cause of swimmer’s itch in Europe, especially for immunocompromised people [10, 47]. As one of the nasal avian schistosomes, *T. regenti* feeds on nervous tissue while migrating through the central nervous system, frequently causing leg paralysis in avian and murine models [48, 49]. Experimental infections in mice show that the immune response in the skin can halt 90% of the schistosomula, but the remaining 10% can reach the spinal cord [9, 50]. Thus far no human neuropathogenic cases linked to *T. regenti* infections have been reported, but a better understanding among medical professionals about this possibility is desirable [47].

Improved public awareness and a centralized contact point to report such cases could aid in the identification of transmission sites. Suspected transmission areas could then be investigated through targeted malacological and parasitological surveys. The use of an integrative taxonomic approach would lead to improved resolution at the species level [51] and thus more precise diagnosis and possibly also better control measures [52]. At the same time, this integrative taxonomic approach would map biodiversity and feed the incomplete reference sequence database of trematodes, a grossly neglected group in barcoding databases [53–55]. Given the paucity of information for trematodes, it is not surprising that our results showcase the troublesome barcoding efforts for Belgian representatives, especially since there is little to no monitoring of trematode diversity in the country. Such a barcoding gap is problematic as it inhibits fast and accurate epidemiological studies due to incomplete disease transmission patterns [53]. To do so, a parasite’s lifecycle should be fully known, linking intermediate snail hosts to final hosts, a piece of the puzzle still lacking for several *Trichobilharzia* species [3, 55]. Since cercarial traits frequently do not provide taxonomic information beyond the family level, molecular tools are imperative to identify both the snail intermediate host and the parasite infecting them [3, 55]. Ideally, this includes the study of adult parasites from the final bird species. Natural history collections could provide a more sustainable alternative, although curated helminth samples have frequently been stored in formaldehyde, a medium complicating DNA sequencing [27, 56, 57]. There are also protocols and even commercial kits for environmental DNA detection of *Trichobilharzia* spp. in water bodies [58–61], which could complement or replace the rather time-consuming malacological and parasitological surveys.

Historic and current reports underline the effect of human environmental changes on trematodiasis outbreaks [27, 62–65]. We argue that the sudden appearance of *T. regenti* in the study area may be attributable to global change and local environmental change. Global change affects snail–trematode interactions [66], bird migration patterns [67], the introduction of invasive species [68], and snail ranges [69], all potentially influencing swimmer’s itch epidemiology. As thoroughly discussed by Soldánová et al. [4] and Mas-Coma et al. [12], climate change can drive the emergence and severity of trematode transmission foci since increased water temperatures result in increased parasite development and cercarial emergence while also increasing human water contact frequency and duration. Water contact intensity has already been linked to increased cercarial dermatitis incidence in the Netherlands [58] and beyond, including the USA [70] and Switzerland [71].

## Conclusions

With the ﻿projected future temperature increase, swimmer’s itch incidence will most likely also continue to increase [3, 4, 11, 12, 72]. We suggest that an improved patient reporting system combined with transmission site localization and monitoring efforts, such as in the Netherlands [36, 73] and Canada [74], will prove invaluable assets to prepare for potential snail-borne parasite outbreaks in Belgium.

### Supplementary Information

**Additional file 1: Table S1**. Table reporting the primers involved for the amplification of the five DNA regions (two and three genes within the snails and the trematodes, respectively). **Table S2**. PCR cycling conditions used for the amplification of the five DNA regions. *The elongation time for our protocol was increased to 60 s compared with the 45 s of Schols et al. [37].

## Data Availability

The resulting sequences of the study are deposited on NCBI GenBank, with accession codes PP203049- PP203073; PP232101- PP232105; and PP274989- PP274991. Tissue, shell, and DNA vouchers are stored in the collection of RBINs with accession codes RBINS:IG34767:INV311000.

## References

[1] Horák P, Mikeš L, Panská L, Skála V, Soldánová M, Brant S (2015). Avian Schistosomes and outbreaks of Cercarial dermatitis. Clin Microbiol Rev.

[2] WHO. Schistosomiasis [fact sheet]. 2023. https://www.who.int/news-room/fact-sheets/detail/schistosomiasis#:~:text=Schistosomiasis. Accessed 27 Nov 2023.

[3] Loker ES, Dejong RJ, Brant SV (2022). Scratching the itch: updated perspectives on the schistosomes responsible for swimmer’s itch around the world. Pathogens.

[4] Soldánová M, Selbach C, Kalbe M, Kostadinova A, Sures B (2013). Swimmer’s itch: etiology, impact, and risk factors in Europe. Trends Parasitol.

[5] Brant SV, Jouet D, Ferte H, Loker ES (2013). *Anserobilharzia* gen. n. (Digenea, Schistosomatidae) and redescription of *A. brantae* (Farr & Blankemeyer, 1956) comb n. (syn. *Trichobilharzia brantae*), a parasite of geese (Anseriformes). Zootaxa.

[6] Poulin R (2006). Global warming and temperature-mediated increases in cercarial emergence in trematode parasites. Parasitology.

[7] Soldánová M, Selbach C, Sures B (2016). The early worm catches the Bird? Productivity and patterns of *Trichobilharzia szidati* cercarial emission from *Lymnaea stagnalis*. PLoS ONE.

[8] Macháček T, Turjanicová L, Bulantová J, Hrdý J, Horák P, Mikeš L (2018). Cercarial dermatitis: a systematic follow-up study of human cases with implications for diagnostics. Parasitol Res.

[9] Hrádková K, Horák P (2002). Neurotropic behaviour of *Trichobilharzia regenti* in ducks and mice. J Helminthol.

[10] Kouřilová P, Syrůček M, Kolářová L (2004). The severity of mouse pathologies caused by the bird schistosome *Trichobilharzia regenti* in relation to host immune status. Parasitol Res.

[11] Christiansen AØ, Olsen A, Buchmann K, Kania PW, Nejsum P, Vennervald BJ (2016). Molecular diversity of avian schistosomes in Danish freshwater snails. Parasitol Res.

[12] Mas-Coma S, Valero MA, Bargues MD (2009). Climate change effects on trematodiases, with emphasis on zoonotic fascioliasis and schistosomiasis. Vet Parasitol.

[13] Scott ME (2023). Helminth-host-environment interactions: looking down from the tip of the iceberg. J Helminthol.

[14] Sousa WP (1992). Interspecific interactions among larval trematode parasites of freshwater and marine snails. Am Zool.

[15] Brant SV, Loker ES (2013). Discovery-based studies of schistosome diversity stimulate new hypotheses about parasite biology. Trends Parasitol.

[16] Caron Y, Cabaraux A, Marechal F, Losson B (2017). Swimmer’s Itch in Belgium: first recorded outbreaks, molecular identification of the parasite species and intermediate hosts. Vector Borne Zoonotic Dis.

[17] De Liberato C, Berrilli F, Bossù T, Magliano A, Montalbano Di Filippo M, Di Cave D, Sigismondi M, Cannavacciuolo A, Scaramozzino P (2019). Outbreak of swimmer’s itch in Central Italy: description, causative agent and preventive measures. Zoonoses Public Health.

[18] Müller V, Kimmig P (1994). *Trichobilharzia franki* n. sp. the cause of swimmer’s dermatitis in southwest German dredged lakes. Appl Parasitol.

[19] Helmer N, Blatterer H, Hörweg C, Reier S, Sattmann H, Schindelar J, Szucsich U, Haring E (2021). First Record of *Trichobilharzia physellae* (Talbot, 1936) in Europe, a possible causative agent of cercarial dermatitis. Pathogens.

[20] Kolářová L, Skírnisson K, Ferté H, Jouet D (2013). *Trichobilharzia mergi* sp. Nov. (Trematoda: Digenea: Schistosomatidae), a visceral schistosome of *Mergus serrator* (L.) (Aves: Anatidae). Parasitol Int..

[21] Jouet D, Kolářová L, Patrelle C, Ferté H, Skírnisson K (2015). *Trichobilharzia anseri* n. sp. (Schistosomatidae: Digenea), a new visceral species of avian schistosomes isolated from greylag goose (*Anser anser* L.) in Iceland and France. Infect Genet Evol..

[22] Simon-Vicente F, Simon-Martin F (1999). The life cycle of *Trichobilharzia salmanticensis* n. sp. (Digenea: Schistosomatidae), related to cases of human dermatitis. Res Rev Parasitol.

[23] Picard D, Jousson O (2001). Genetic variability among cercariae of the Schistosomatidae (Trematoda: Digenea) causing swimmer’s itch in Europe. Parasite.

[24] Jansen B (2015). Veldgids Slakken en Mossels.

[25] Frandsen F, Christensen NØ (1984). An introductory guide to the identification of cercariae from African freshwater snails with special reference to cercariae of trematode species of medical and veterinary importance. Acta Trop.

[26] Brecko J, Mathys A, Dekoninck W, Leponce M, VandenSpiegel D, Semal P (2014). Focus stacking: comparing commercial top-end set-ups with a semi-automatic low budget approach. A possible solution for mass digitization of type specimens. Zookeys.

[27] Schols R, Carolus H, Hammoud C, Muzarabani KC, Barson M, Huyse T (2021). Invasive snails, parasite spillback, and potential parasite spillover drive parasitic diseases of *Hippopotamus amphibius* in artificial lakes of Zimbabwe. BMC Biol.

[28] Zietara MS, Arndt A, Geets A, Hellemans B, Volckaert FA (2000). The nuclear rDNA region of *Gyrodactylus arcuatus* and *G. branchicus* (Monogenea: Gyrodactylidae). J Parasitol.

[29] Lobo J, Costa PM, Teixeira MAL, Ferreira MSG, Costa MH, Costa FO (2013). Enhanced primers for amplification of DNA barcodes from a broad range of marine metazoans. BMC Ecol.

[30] Vinarski M, Schniebs K, Glöer P, Hundsdoerfer A (2011). The taxonomic status and phylogenetic relationships of the genus *Aenigmomphiscola* Kruglov and Starobogatov, 1981 (Gastropoda: Pulmonata: Lymnaeidae). J Nat Hist.

[31] Schniebs K, Glöer P, Vinarski M, Hundsdoerfer A (2016). A barcode pitfall in Palaearctic *Stagnicola* specimens (Mollusca: Lymnaeidae): Incongruence of mitochondrial genes, a nuclear marker and morphology. North West J Zool.

[32] Carolus H, Muzarabani KC, Hammoud C, Schols R, Volckaert FAM, Barson M, Huyse T (2019). A cascade of biological invasions and parasite spillback in man-made Lake Kariba. Sci Total Environ.

[33] Hammoud C, Mulero S, Boissier J, Van Bocxlaer B, Verschuren D, Albrecht C, Huyse T (2022). Simultaneous genotyping of snails and infecting trematode parasites using high-throughput amplicon sequencing. Mol Ecol Resour.

[34] Littlewood DTJ, Waeschenbach A, Nikolov PN (2008). In search of mitochondrial markers for resolving the phylogeny of cyclophyllidean tapeworms (Platyhelminthes, Cestoda) — a test study with Davaineidae. Acta Parasitol.

[35] Schols R, Vanoverberghe I, Huyse T, Decaestecker E (2023). Host-bacteriome transplants of the schistosome snail host *Biomphalaria glabrata* reflect species-specific associations. FEMS Microbiol Ecol.

[36] Gittenberger E, Janssen AW, Kuijper WJ, Kuiper JG, Meijer T, van der Velde G, et al. De Nederlandse zoetwatermollusken. Recente en fossiele weekdieren uit zoet en brak water. Nationaal Natuurhistorisch Museum, Naturalis, Nederland; 1998.

[37] Ebbs ET, Loker ES, Brant SV (2018). Phylogeography and genetics of the globally invasive snail Physa acuta Draparnaud 1805, and its potential to serve as an intermediate host to larval digenetic trematodes. BMC Evol Biol.

[38] Toledo R, Fried B (2014). Digenetic Trematodes.

[39] Boyce K, Craig PS, Harris PD, Hide G, Pickles A, Reynolds C, Rogan M T (2012). Identification of a new species of digenean *Notocotylus malhamensis* n. sp. (Digenea: Notocotylidae) from the bank vole (*Myodes glareolus*) and the field vole (*Microtus agrestis*). Parasitology.

[40] Horák P, Kolářová L, Dvořák J (1998). *Trichobilharzia regenti* n. sp. (Schistosomatidae, Bilharziellinae), a new nasal schistosome from Europe. Parasite..

[41] Jouet D, Skírnisson K, Kolářová L, Ferté H (2010). Final hosts and variability of *Trichobilharzia regenti* under natural conditions. Parasitol Res.

[42] Marszewska A, Strzała T, Cichy A, Dąbrowska GB, Żbikowska E (2018). Agents of swimmer’s itch—dangerous minority in the Digenea invasion of Lymnaeidae in water bodies and the first report of *Trichobilharzia regenti* in Poland. Parasitol Res.

[43] Rudolfova J, Littlewood DTJ, Sitko J, Horak P (2007). Bird schistosomes of wildfowl in the Czech Republic and Poland. Folia Parasitol (Praha).

[44] Jouet D, Ferté H, Depaquit J, Rudolfová J, Latour P, Zanella D, Kaltenbach ML, Léger N (2008). *Trichobilharzia* spp. in natural conditions in Annecy Lake, France. Parasitol Res.

[45] Al-Jubury A, Buchmann K, Bygum A, Duan Y, Horák P, Jørgensen LVG, Kania PW, Tracz ES (2021). Avian schistosome species in Danish freshwater lakes: relation to biotic and abiotic factors. J Helminthol.

[46] Lopatkin AA, Khrisanfova GG, Voronin MV, Zazornova OP, Beér SA, Semenova SK (2010). Polymorphism of the cox1 gene in bird schistosome cercaria isolates (Trematoda, Schistosomatidae) from ponds of Moscow and Moscow oblast. Genetika.

[47] Lichtenbergová L, Horák P (2012). Pathogenicity of *Trichobilharzia* spp. for Vertebrates. J Parasitol Res.

[48] Horák P, Dvořák J, Kolářová L, Trefil L (1999). *Trichobilharzia regenti*, a pathogen of the avian and mammalian central nervous systems. Parasitology.

[49] Macháček T, Leontovyč R, Šmídová B, Majer M, Vondráček O, Vojtěchová I, Petrásek T, Horák P (2022). Mechanisms of the host immune response and helminth-induced pathology during *Trichobilharzia regenti* (Schistosomatidae) neuroinvasion in mice. PLoS Pathog.

[50] Macháček T, Šmídová B, Pankrác J, Majer M, Bulantová J, Horák P (2020). Nitric oxide debilitates the neuropathogenic schistosome *Trichobilharzia regenti* in mice, partly by inhibiting its vital peptidases. Parasit Vectors.

[51] Will KW, Mishler BD, Wheeler QD (2005). The perils of DNA barcoding and the need for integrative taxonomy. Syst Biol.

[52] Blasco-Costa I, Cutmore SC, Miller TL, Nolan MJ (2016). Molecular approaches to trematode systematics: ‘best practice’ and implications for future study. Syst Parasitol.

[53] Kvist S (2013). Barcoding in the dark?: A critical view of the sufficiency of zoological DNA barcoding databases and a plea for broader integration of taxonomic knowledge. Mol Phylogenet Evol.

[54] Schols R, Mudavanhu A, Carolus H, Hammoud C, Muzarabani KC, Barson M, Huyse T (2020). Exposing the barcoding void: an integrative approach to study snail-borne parasites in a one health context. Front Vet Sci.

[55] Brant SV, Morgan JAT, Mkoji GM, Snyder SD, Rajapakse RPVJ, Loker ES (2006). An approach to revealing blood fluke life cycles, taxonomy, and diversity: provision of key reference data including DNA sequence from single life cycle stages. J Parasitol.

[56] Shedlock AM, Haygood MG, Pietsch TW, Bentzen P (1997). Enhanced DNA extraction and PCR amplification of mitochondrial genes from formalin-fixed museum specimens. Biotechniques.

[57] Herrmann B, Hummel S (1994). Ancient DNA: recovery and analysis of genetic material from paleontological, archaeological, museum, medical, and forensic specimens.

[58] Schets FM, Lodder WJ, van Duynhoven YTHP, de Roda Husman AM (2008). Cercarial dermatitis in the Netherlands caused by *Trichobilharzia* spp. J Water Health.

[59] Schets FM, Lodder WJ, de Roda Husman AM (2010). Confirmation of the presence of *Trichobilharzia* by examination of water samples and snails following reports of cases of cercarial dermatitis. Parasitology.

[60] Jothikumar N, Mull BJ, Brant SV, Loker ES, Collinson J, Secor WE, Hill VR (2015). Real-time PCR and sequencing assays for rapid detection and identification of avian schistosomes in environmental samples. Appl Environ Microbiol.

[61] Rudko SP, Reimink RL, Froelich K, Gordy MA, Blankespoor CL, Hanington PC (2018). Use of qPCR-based cercariometry to assess swimmer’s itch in recreational lakes. EcoHealth.

[62] Sow S, de Vlas SJ, Engels D, Gryseels B (2002). Water-related disease patterns before and after the construction of the Diama dam in northern Senegal. Ann Trop Med Parasitol.

[63] Halstead NT, Hoover CM, Arakala A, Civitello DJ, De Leo GA, Gambhir M, Johnson SA, Jouanard N, Loerns KA, McMahon TA, Ndione RA, Nguyen K, Raffel TR, Remais JV, Riveau G, Sokolow SH, Rohr JR (2018). Agrochemicals increase risk of human schistosomiasis by supporting higher densities of intermediate hosts. Nat Commun.

[64] Sokolow SH, Jones IJ, Jocque M, La D, Cords O, Knight A, Lund A, Wood CL, Lafferty KD, Hoover CM, Collender PA, Remais JV, Lopez-Carr D, Fisk J, Kuris AM, De Leo GA (2017). Nearly 400 million people are at higher risk of schistosomiasis because dams block the migration of snail-eating river prawns. Phil Trans Royal Soc B Biol Sci.

[65] Hira PR (1969). Transmission of Schistosomiasis in Lake Kariba. Zambia Nature.

[66] Paull SH, Johnson PTJ (2011). High temperature enhances host pathology in a snail–trematode system: possible consequences of climate change for the emergence of disease. Freshw Biol.

[67] Jenni L, Kéry M (2003). Timing of autumn bird migration under climate change: advances in long–distance migrants, delays in short–distance migrants. Proc R Soc London Ser B Biol Sci.

[68] Dukes JS, Mooney HA (1999). Does global change increase the success of biological invaders?. Trends Ecol Evol.

[69] Cordellier M, Pfenninger A, Streit B, Pfenninger M (2012). Assessing the effects of climate change on the distribution of pulmonate freshwater snail biodiversity. Mar Biol.

[70] Verbrugge L, Rainey J, Reimink R, Blankespoor H (2004). Prospective study of swimmer’s itch incidence and severity. J Parasitol.

[71] Chamot E, Toscani L, Rougemont A (1998). Public health importance and risk factors for cercarial dermatitis associated with swimming in Lake Leman at Geneva. Switzerland Epidemiol Infect.

[72] Kaffenberger BH, Shetlar D, Norton SA, Rosenbach M (2017). The effect of climate change on skin disease in North America. J Am Acad Dermatol.

[73] de Lange HJ, Bijkerk R, de Groot GA. Zwemmersjeuk in Nederland: resultaten van een meta-analyse naar vóórkomen zwemmersjeuk en mogelijkheden voor een effectieve aanpak. Stowa; 2017.

[74] Gordy MA, Cobb TP, Hanington PC (2018). Swimmer’s itch in Canada: a look at the past and a survey of the present to plan for the future. Environ Heal.

