# Supplementary material for: Expanding the swimmer’s itch pool of the Benelux: a first record of the neurotropic Trichobilharzia regenti and potential link to human infection
Source: Parasit Vectors. 2024 Mar 13;17:126. doi: 10.1186/s13071-024-06218-4 (PMC10938770; doi:10.1186/s13071-024-06218-4)
Supplement: Supplementary file 1 — Additional file 1: Table S1. Table reporting the primers involved for the amplification of the five DNA regions (two and three genes within the snails and the trematodes, respectively). Table S2. PCR cycling conditions used for the amplification of the five DNA regions. *The elongation time for our protocol was increased to 60 s compared with the 45 s of Schols et al. [37]. [file 13071_2024_6218_MOESM1_ESM.docx]

**Additional file 1:** DNA amplification protocol. **Table S1**: Table reporting the primers involved for the amplification of the five DNA regions (two and three genes within the snails and the trematodes, respectively). **Table S2**: PCR cycling conditions used for the amplification of the five DNA regions.*The elongation time for our protocol was increased to 60 seconds compared to the 45 seconds of Schols et al. [37].

**Table S1**

| Target taxa | Target gene | Primer combinations | | | | | | PCR profile ID (see Table S2) |
| --- | --- | --- | --- | --- | --- | --- | --- | --- |
|  |  | **Forward** | | | **Reverse** | | |  |
|  |  | **Name** | **Sequence** | **Reference** | **Name** | **Sequence** | **Reference** |  |
| Snail | COI | LoboF1 | kbtchacaaaycayaargayathgg | Lobo *et al.* [1] | LoboR1 | taaacytcwggrtgwccraaraayca | Lobo *et al.* [1] | PCR_Snail_COI |
|  | ITS2 | LT1 | TCGTCTGTGTGAGGGTCG | Bargues *et al.* [2] | ITS2_Rixo | TTCTATGCTTAAATTCAGGGG | Almeyda-Artigas *et al.* [3] | PCR_Snail_ITS2 |
| Trematode | COI | COI1_Dig_F | CNATGATNTTNTTTTTTTTRATGCC | Carolus *et al.* [4] | COI2_tremR | ACATAATGAAARTGAGCNAYMACRA | Hammoud *et al.* [5] | PCR_Trematode_COI_1 |
|  |  | COI1_Dig_F | CNATGATNTTNTTTTTTTTRATGCC | Carolus *et al.* [4] | COI1_DIG_R | GMASWACCAAAWTTHCGATCAAA | Carolus *et al.* [4] | PCR_Trematode_COI_2 & ITS2 |
|  | ITS2 | ITS2_tremF | CAAHAAGTCGTGGMTTGG | Hammoud *et al.* [5] | ITS2_tremR | CCTAAACACCACATTGCCT | Hammoud *et al*. [5] |  |
|  | 18S | 18S_Digenea_F | CAGCTATGGTTCCTTAGATCRT | Carolus *et al.* [4] | 1270R | CCGTCAATTCCTTTAAGT | Littlewood *et al.* [6] | PCR_Trematode_18S |

**Table S2**

| PCR_profile_ID (refer to Table S1) | Initial denaturation | Denaturation | Annealing | Elongation | Final elongation | Cycles | Reference |
| --- | --- | --- | --- | --- | --- | --- | --- |
| PCR_Snail_COI | 94°C - 3 min | 94°C - 30 sec | 45 °C - 90 sec | 72 °C - 60 sec | / | 5 | Lobo *et al.* [1] |
|  | / | 94 °C - 30 sec | 54 °C - 90 sec | 72 °C - 60 sec | 72 °C - 10 min | 45 |  |
| PCR_Snail_ITS2 | 94°C - 5 min | 94°C - 30 sec | 50°C - 30 sec | 72°C - 45 sec | 72°C - 10 min | 40 | Schols *et al.* [7] based on [8], [9] |
| PCR_Trematode_COI_1 | 94 °C - 3 min | 94 °C - 30 sec | 53 °C - 45 sec | 72 °C - 60 sec | 72 °C - 10 min | 40 | Schols *et al*. [10]* |
| PCR_Trematode_COI_2 & ITS2 | 94 °C - 3 min | 94 °C - 30 sec | 50 °C - 45 sec | 72 °C - 60 sec | 72 °C - 10 min | 40 |  |
| PCR_Trematode_18S | 94 °C - 3 min | 94 °C - 30 sec | 50 °C - 90 sec | 72 °C - 60 sec | 72 °C - 10 min | 40 |  |

[1] J. Lobo, P. M. Costa, M. A. L. Teixeira, M. S. G. Ferreira, M. H. Costa, and F. O. Costa, “Enhanced primers for amplification of DNA barcodes from a broad range of marine metazoans,” *BMC Ecol.*, vol. 13, no. 1, p. 34, 2013, doi: 10.1186/1472-6785-13-34.

[2] M. D. Bargues *et al.*, “European Lymnaeidae (Mollusca: Gastropoda), intermediate hosts of trematodiases, based on nuclear ribosomal DNA ITS-2 sequences,” *Infect. Genet. Evol.*, vol. 1, no. 2, pp. 85–107, 2001, doi: https://doi.org/10.1016/S1567-1348(01)00019-3.

[3] R. J. Almeyda-Artigas, M. D. Bargues, and S. Mas-Coma, “ITS-2 rDNA sequencing of Gnathostoma species (Nematoda) and elucidation of the species causing human gnathostomiasis in the Americas.,” *J. Parasitol.*, vol. 86, no. 3, pp. 537–544, Jun. 2000, doi: 10.1645/0022-3395(2000)086[0537:IRSOGS]2.0.CO;2.

[4] H. Carolus *et al.*, “A cascade of biological invasions and parasite spillback in man-made Lake Kariba,” *Sci. Total Environ.*, vol. 659, pp. 1283–1292, 2019, doi: 10.1016/j.scitotenv.2018.12.307.

[5] C. Hammoud *et al.*, “Simultaneous genotyping of snails and infecting trematode parasites using high-throughput amplicon sequencing,” *Mol. Ecol. Resour.*, vol. 22, no. 2, pp. 567–586, 2022, doi: 10.1111/1755-0998.13492.

[6] D. T. J. Littlewood, A. Waeschenbach, and P. N. Nikolov, “In search of mitochondrial markers for resolving the phylogeny of cyclophyllidean tapeworms (Platyhelminthes, Cestoda) — a test study with Davaineidae,” *Acta Parasitol.*, vol. 53, no. 2, pp. 133–144, 2008, doi: 10.2478/s11686-008-0029-4.

[7] R. Schols, I. Vanoverberghe, T. Huyse, and E. Decaestecker, “Host-bacteriome transplants of the schistosome snail host Biomphalaria glabrata reflect species-specific associations,” *FEMS Microbiol. Ecol.*, vol. 99, no. 9, p. fiad101, 2023.

[8] K. Schniebs, P. Glöer, M. Vinarski, and A. Hundsdoerfer, “A barcode pitfall in Palaearctic Stagnicola specimens (Mollusca: Lymnaeidae): Incongruence of mitochondrial genes, a nuclear marker and morphology,” *North. West. J. Zool.*, vol. 12, pp. 239–254, Dec. 2016.

[9] M. Vinarski, K. Schniebs, P. Glöer, and A. Hundsdoerfer, “The taxonomic status and phylogenetic relationships of the genus *Aenigmomphiscola* Kruglov and Starobogatov, 1981 (Gastropoda: Pulmonata: Lymnaeidae),” *J. Nat. Hist.*, vol. 45, pp. 2049–2068, Sep. 2011, doi: 10.1080/00222933.2011.574800.

[10] R. Schols, H. Carolus, C. Hammoud, K. C. Muzarabani, M. Barson, and T. Huyse, “Invasive snails, parasite spillback, and potential parasite spillover drive parasitic diseases of Hippopotamus amphibius in artificial lakes of Zimbabwe,” *BMC Biol.*, vol. 19, no. 1, p. 160, 2021, doi: 10.1186/s12915-021-01093-2.
